# Household energy-saving behavior, its consumption, and life satisfaction in 37 countries

**DOI:** 10.1038/s41598-023-28368-8

**Published:** 2023-01-25

**Authors:** Xiangdan Piao, Shunsuke Managi

**Affiliations:** 1 Faculty of Humanities and Social Science, Iwate University, 3-18-34 Ueda, Morioka, Iwate 020-8550 Japan; 2 Urban Institute & School of Engineering, Kyushu University, 744 Motooka Nishi-Ku, Fukuoka, 819-0395 Japan

**Keywords:** Ecology, Environmental social sciences

## Abstract

Since energy consumption became an important contributor to climate change owing to carbon emissions, energy-saving behavior and expenditure at the household level have been attracting scholars’ and policymakers’ attention. This study identified whether greenhouse gas emissions at the household level can be reduced through purchase of energy-saving goods and whether the energy-saving behavior enhanced with household income increase. We conducted a large-scale survey across 37 nations using internet-based and face-to-face approaches, collecting 100,956 observations. The wealth effect on energy consumption expenditure at the household level was found to be positive across countries, confirming that energy consumption increases with household wealth improvement. Furthermore, households show a positive association between household energy expenditure and life satisfaction in 27 out of 37 countries, including China, India, the United States, and Germany. Additionally, the favorable effects of household energy-saving behavior are confirmed. However, purchase of household energy-saving products has a limited effect on energy consumption expenditure, compared with that of energy-curtailment behavior. In conclusion, achieving a carbon–neutral household by reducing energy consumption expenditure at the household level is challenging; thus, along with the use of energy-saving goods, alternative energy sources, such as renewable energies, are recommended.

## Introduction

Energy consumption is closely related to global climate change through greenhouse gas emissions. Hence, enhancing humanity’s well-being via sustainable energy consumption and environmental conservation is crucial. In this study, we aim to identify whether greenhouse gas emissions at the household level can be reduced by reducing the energy consumption expenditure of households globally. In 2015, the United Nations proposed the sustainable development goals for sustaining humanity’s well-being, encompassing 17 multidimensional goals related to environment preservation, economics, and society. Subjective well-being is assumed to be a proxy for humanity’s well-being both in sociological and other psychological and economic aspects^1–3^.

Since the Industrial Revolution, fossil fuels, which include natural gas, coal, and oil, have become a crucial energy source for modern industries. As fossil fuel consumption is associated with greenhouse gas emissions, including carbon dioxide (CO2) emissions, global CO2 emissions from fossil fuels increased from 14 billion tons in 1971 to 34 billion tons in 2016^4^. The Fifth Assessment Report of climate change released in 2013 concludes that global warming is undoubtedly caused by human activities. The Paris agreement sets a clear goal to “limit global warming to well below 2, preferably to 1.5 degrees Celsius compared to pre-industrial levels”^5^. To achieve this goal, policies to reduce CO2 emissions were introduced across the globe. For example, according to the IEA^6^, in-building light, space heating, and water heating increased to 83%, 43%, and 39%, respectively, in 2018. Furthermore, the transition to zero-emission vehicles was announced in Europe, Asia, and the Americas^7^. Additionally, efficiency stars were initiated for electronic products to meet the energy efficiency standards of the United States Environmental Protection Agency and Department of Energy.

Numerous studies have examined the association between well-being and energy consumption, with inconclusive results^8–18^. Chapman et al.^13^ used individual-level micro-cross-sectional data from 37 nations to demonstrate that households often have difficulties in terms of being able to afford the costs of energy consumption and that individuals from such households are more likely to experience a lower quality of life. Niu et al.^9^ used country-level panel data from 50 countries to describe the positive causal effect of energy consumption and human development in these countries; the authors also encouraged governments to provide low-income residential electricity as public services. By contrast, using country-level panel data, Mazur^8^ argued that the associations between energy, electricity consumption, and quality of life improvements are not significant. The author also stated that the significant association between these variables may originate from analyses of cross-sectional data at the country level. Jorgenson et al.^11^ also discussed the relationship between energy intensity and human well-being, particularly within the context of central and eastern European nations; these authors found that the relationship between these two variables in these two contexts is rather complex and is undergoing dramatic changes.

Energy-saving behavior belongs to the category of pro-environmental behavior, with the latter being defined as altruistic, friendly, and contributive behavior toward environmental conservation^19–39^. In this study, energy-saving behaviors refer to those that reduce overall energy usage, including electricity and fuels^21,22^. To quantify the different types of energy-saving behavior, we adopt energy curtailment behavior and purchasing energy-saving goods as well as household energy efficiency behavior. Energy curtailment behavior is the low financial cost of energy consumption reduction behaviors, such as turning off power to appliances when not in use. Purchase of energy-saving goods reflect household energy efficiency as it reduces the high cost of energy consumption. Examples of variables used as proxies of energy-saving behavior are recycling, reuse, and energy-saving behavior in selection of the means of transportation^27–39^. Substantial literature has investigated the determinants of pro-environmental behavior and found the following key factors: knowledge of environmental issues, environmental experiences at a young age, culture, consumption beliefs, and psychological factors^29–40^.

Various studies show that unbridled energy consumption can be a threat to the environment^14,16–20^. Moreover, scholars and policymakers have been focusing their attention on the impact of household consumption. For example, the Japanese government has set up a goal for the household sector to reduce 66% of its CO2 emissions by 2030 to ensure the achievement of the nation’s greenhouse gas emissions reduction goals. Nonetheless, according to traditional economic theories and the subjective well-being framework, households consume energy within the context of their wealth constraints and aim to maximize the utility of the consumed energy. Subjective well-being has been described in past research as a useful measurement for assessing people’s well-being. Theoretical and empirical findings provide conflicting evidence on the association between environmental conservation goals and hedonic goals^32,33,35,41–43^.

When energy is seen as a consumption good, energy consumption expenditure may increase as household income increases, indicating a positive relationship between household income and energy consumption expenditure. The key energy consumption metric is the quantity of energy consumed (e.g., kWh) across the targeted households. Since price information is limited, transforming consumption expenditure into a quantity (e.g., kWh) is problematic. According to the theory, measurement issues are common in that household expenditure measurement is based on the expenditure amount rather than the quantity consumed. Theoretically and empirically, studies have tried to address this measurement issue by estimating the demand system^44–48^. Although the information relates to quantity, the observable measurement is the expenditure. Recently, Du et al.^47^ estimated the energy demand function based on the demand system model. Thus, following previous research, the present study adopted this method of estimating using the energy demand equation. However, the goal framing theory presents the individual’s involvement in pro-environment behavior, that is, energy-saving behavior, under the normative goal, as practicing energy curtailment or energy efficiency behavior is the right thing to do^41,49^.

Moreover, if people are satisfied with their energy consumption, it might be difficult for households to reduce their energy consumption expenditure. In this context, policymakers should consider alternative tools to reduce greenhouse gas emissions at the household level. Investigations into the relationship between energy consumption at the household level and subjective well-being may provide insights as to whether households might be capable of cutting their energy consumption to help reduce greenhouse gas emissions. When the climate change situation is exacerbating, energy saving behavior is expected to sustain the environment. The fossil sourced energy, for example, electricity, curtailment behavior or energy efficiency behavior is proceeded^49–54^. However, research focusing on energy consumption, subjective well-being, and environmental-friendly energy consumption outcomes from a multi-national level perspective remain scarce. This study aims to address this knowledge gap.

This study contributes to the literature in the following aspects. First, the survey encompasses 37 nations, accounting for approximately 73% of the world’s population, providing data that serve to illustrate the effect of energy consumption expenditure on subjective well-being. The wealth effect is also examined within this context. The results are expected to highlight whether an increase in energy consumption leads to economic development. Second, this study lists the key determinants of energy consumption expenditure in households, providing important data that may have policy implications, such as being used in the simulation of energy consumption at the household level.

The remainder of this paper is structured as follows. Section "Methods" offers the study data and outlines the methodology, Section "Results" reports the results, and Section "Discussion" presents the discussion them. Section "Conclusion" concludes the paper.

## Methods

### Data collection

To explore the relationships between subjective well-being and energy consumption expenditure at the household level, this study conducted a large-scale, original, cross-sectional survey with samples from 37 nations using internet-based and face-to-face approaches. The data collection process was as follows. First, the random sampling process was applied to match the population age and gender characteristics. To do this, based on the gender and age distribution in each nation, the population was divided into numerous groups. Among all age and gender groups, restricted panels of women older than 60 years of age are scarce; therefore, an age group closest to it, that is, 55–59 years of age, was selected to avoid sample selection bias.

Second, the targeted respondents were randomly selected through a reputed company and the questionnaire was distributed to them via the internet. The company has comprehensive registered panels that enhance the collected samples to match the country's specified gender and age distributions. Moreover, the sample collection is conducted among countries separately, and to enhance the reliable of the empirical regression results, the sample size for each country is greater than 500. For each country, the number of observations ranged from 500–20,744, with the total number of observations being 100,956 over 2015–2017 (see Table 1).

**Table 1:** Survey type and number of observations in 37 nations.

Country name	Survey type	Obs	Country name	Survey type	Obs
Japan	Internet	11,167	Egypt	Face to face	1016
Thailand	Internet	1127	Russia	Internet	2221
Malaysia	Internet	1106	China	Internet	20,744
Indonesia	Internet	2210	Australia	Internet	2029
Singapore	Internet	587	United States	Internet	10,683
Vietnam	Internet	1541	Germany	Internet	3165
Philippines	Internet	1686	United Kingdom	Internet	2993
Mexico	Internet	1678	France	Internet	2138
Venezuela	Internet	827	Spain	Internet	2116
Chile	Internet	1192	Italy	Internet	2106
Brazil	Internet	2298	Sweden	Internet	1330
Colombia	Internet	1115	Canada	Internet	1333
South Africa	Internet	1123	Netherlands	Internet	1371
India	Internet	5200	Greece	Internet	1382
Myanmar	Face to face	1083	Turkey	Internet	2120
Indonesia	Face to face	202	Hungary	Internet	1354
Vietnam	Face to face	200	Poland	Internet	2227
India	Face to face	1500	Czech Republic	Internet	1400
Kazakhstan	Face to face	1000	Romania	Internet	1386
Mongolia	Face to face	500	Sri Lanka	Face to face	500

Third, because internet users tend to be younger and more well-educated than non-internet users, the internet survey was likely to select individuals with better wealth status and good education level^55^. To counter this potential sample collection issue, the internet-based survey covered 32 nations, and the face-to-face survey was conducted in Mongolia, Myanmar, Egypt, Kazakhstan, and Sri Lanka, wherein the application of an internet-based survey was considered. Both types of surveys were conducted in Indonesia, India, and Vietnam. When conducting the face-to-face survey, survey agents visited the targeted area to collect data directly in the field along with the coauthor of this study. The agents were given extensive training. Although in the face-to-face survey, the random sampling process was not followed, the sample is valuable to present households’ energy consumption situation among rural or slum areas. Furthermore, the questionnaire was translated and repeatedly checked by professional translators to enhance accuracy. The internet-based survey covered 32 nations. The targeted countries were selected based on their regional representative population size, development representative economies, as well as cultural representativeness, that is, China and Japan are highly influenced by Confucianism, whereas Western countries share individualism, religion, and social norms.

The survey was designed to collect individuals’ perceived satisfaction in their lives, concerns about the environment, cooperation in energy usage that can be seen as energy-saving behavior, household income, energy expenditure, and other households’ demographics and economic background. In the choice items’ design for sensitive questions (e.g., household income), the exclusive items or “do not know” or unlikely-to-answer items were added to avoid dropout by respondents and improve the accuracy of the data. The survey type and number of observations in 37 nations are displayed in Table 1.

### Variable setting

Life satisfaction is a dependent variable based on the Organization of Economic Cooperation and Development guidelines^56^. When policymakers aim to improve citizens’ well-being, the individual well-being level is unobservable. Therefore, subjective well-being is adopted to reflect citizens’ well-being. In measuring subjective well-being, life satisfaction and happiness are utilized in the literature, and the Cantrill ladder that measure the overall satisfaction is widely adopted^57–59^. The robustness check is applied for happiness, a way to measure individual subjective well-being.

To measure life satisfaction, we asked respondents to answer the following question: “Please imagine a ladder with steps numbered 0–10. The top and bottom of the ladder represent the best and worst possible lives for you, respectively. On which step of the ladder would you say you personally feel you currently stand? (10 = Best possible life; and 0 = Worst possible life).” Regarding happiness levels, the respondents were asked, “Overall, how happy are you with your life?” The response scale ranged from 1–5 (1, *unhappy*; 2, *slightly unhappy*; 3, *neither*; 4, *slightly happy*; 5, *very happy*).

Energy consumption expenditure at the household level was converted into US dollars (USD) for all countries and categorized as the energy-consumption share of the monthly income and household income (the exchange rate is of January 7, 2021). In particular, for energy consumption measurement, the respondents were asked, “What is the average share of the energy bill (including a charge for electricity/ gas/ water/ kerosene/ gasoline) out of your monthly income?” The choices were: do not use at all = 1, 1–9% = 2, 10–19% = 3, 20–29% = 4, 30–39% = 5, 40–49% = 6, 50% and above = 6. To reduce missing value observations, we added the “do not know” choice. The household energy consumption expenditure was converted based on the categorized energy consumption and monthly household income. The subjective price of energy was measured as follows: we asked respondents the following question: “How do you feel about electricity/gas/water/kerosene/gasoline bills? Please select an item that best describes your thoughts.” The response scale ranged from 1–6 (6, *very expensive*; 5, *slightly expensive*; 4, *just right*; 3, *slightly cheap*; 2, *very cheap*; 1, *do not care*; 0, *do not use at all*). The subjective price was calculated equally for all energy categories (i.e., electricity, gas, water, kerosene, and gasoline bills).

The dummy variables of energy-saving behaviors include (1) energy-curtailment behaviors (e.g., saving electricity, fuel, etc.); and (2) purchasing energy-saving household products. Other control variables include household income, educational attainment, age, occupational status, household status, number of children, and gender dummy.

### Methodology

To investigate the relationship between household energy consumption, subjective well-being, and the determinants of energy consumption expenditure at the household level, both Eqs. (1) and (2) were estimated using the ordered logit model^60,61^. The ordered probit, ordered logit, and ordinary least square (OLS) models are considered appropriate when the independent variable is ordinal^60,61^; therefore, the ordered probit and OLS models were used as robustness check. The relationship between household energy consumption and subjective well-being is demonstrated in Eq. (1):

1 $${L}_{iC}=\alpha +{K}_{iC}^{^{\prime}}\theta +{X}_{iC}^{^{\prime}}\beta +{D}_{C}^{^{\prime}}\delta +{\varepsilon }_{iC}$$

where ${L}_{iC}$ denotes the subjective well-being indices (e.g., life satisfaction and happiness levels) of individual $i$ from country $C$. The independent variable,${K}_{iC}$, denotes household energy consumption and is a continuous variable. $X$ is a set of exogenous variables, including the following socioeconomic factors: household income, education attainment, age, occupational status, household status, number of children, and gender dummy. While ${D}_{C}$ is the country dummy in country $C$ used to capture country-level heterogeneity, ${\varepsilon }_{iC}$ is the error term*. *$\alpha$*, *$\theta$*, *$\beta$*,* and $\delta$ are parameters estimated using an ordered logit regression model with Stata 16.

The Likelihood Ratio (***LR***) Chi-Square test and Pseudo R-squared for the ordered logistic regression model and the ordered probit model were applied to measure the goodness of the fit, whereas F-statistics and adjusted R-squared were used for the OLS model.

In Eq. (2), the association of life satisfaction and energy consumption expenditure at the household level for each country was estimated using an ordered logit model, as follows:

2 $${L}_{i}=d+f{K}_{i}+{X}_{i}^{^{\prime}}m+{\varepsilon }_{i}$$

where life satisfaction is the dependent variable (ranging from 0–10, with 0 being the worst and 10 the best possible life). $K$ is a continuous variable for energy consumption expenditure at the household level. Moreover, $X$ denotes socioeconomic and demographic factors. ${\varepsilon }_{i}$ is the error term, and $d$, $f$, and $m$ are the estimated parameters in the ordered logit regression model. All estimations were conducted using Stata 16.

The types of socioeconomic and demographic factors influencing the energy consumption expenditure of households were investigated using Eq. (3) based on energy demand equation and an OLS model^44,62–64^:

3 $${K}_{i}=a+b{Z}_{i}+{M}_{i}^{^{\prime}}c+{\varepsilon }_{i}$$

where the dependent variable, ${K}_{i}$, is energy consumption expenditure at the household level and a continuous variable indicating a larger energy consumption as it increases. $Z$ denotes household income, $M$ denotes energy saving behavior, socioeconomic, demographic factors and other control variables, including subjective price of electricity/gas/water/kerosene/gasoline, household status, age, education attainment, and occupational status. The estimated parameters are $a,b$ and $c$. All estimations were conducted using Stata 16. ${\varepsilon }_{i}$ is the error term. The robustness check was confirmed based on the two-stage least square estimation.

According to household consumption theory, measurement issues are common in that household expenditure measurement is based on the amount (e.g., USD) rather than the quantity consumed (e.g., kWh). Theoretically and empirically, previous studies aimed to address this measurement issue by estimating the demand system^44–48^. Following previous research, this study adopted the energy demand equation method. Consider that the parameter is positive values and statistically significant. In that case, the results indicate that energy is the normal good and that demand for energy consumption increases when household income does. As a robustness check, the different types of the energy demand equation were applied according to previous studies^49–51^.

### Ethics approval and consent to participate

For the original cross-sectional survey conducted by a company (Nikkei Research Company) between 2015 and 2017, the study design was approved by the appropriate legal and ethics review board of Kyushu University. The data were collected with informed consent from participants, following legal and ethical guidelines. All the methods were carried out in accordance with relevant guidelines and regulations of Kyushu University.

## Results

**Figure 1:**
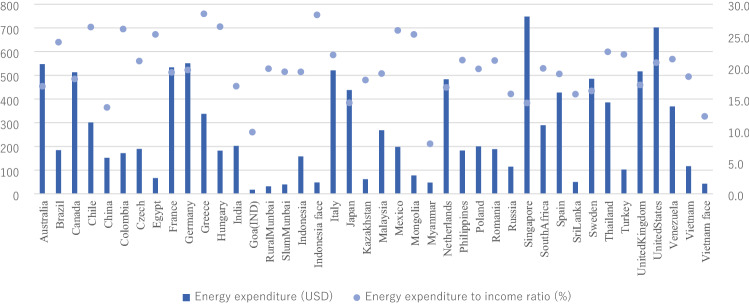
Average monthly energy expenditure at the household level across the 37 surveyed nations. *Data source*: Original survey.

Figure 1 presents the average monthly energy expenditure at the household level based on USD across the 37 surveyed nations. The households in Singapore expend the most amount of energy, that is, 748 USD each month on average. The energy consumption appears positively associated with the economic development level; for example, households from high-income countries, including France, Italy, Japan and the US, tend to consume more energy than those from low-income countries (e.g., Kazakhstan, Myanmar, and Mongolia). In India, Indonesia, and Vietnam, households with higher income expend more on energy than rural/slum households. For the energy expenditure to household income ratio, strong trends were not found between developing and developed countries. Notably, middle-income countries (e.g., Greece, Chile, Brazil, Egypt) spend a relatively higher share of total income on energy.

The relationship between subjective well-being and energy consumption expenditure based on the ordered logit, ordered probit, and OLS models is shown in Table 2, panel A. The ***LR*** Chi-Square test and Pseudo R-squared for the ordered logistic regression model and the ordered probit model were applied to measure the goodness of the fit, whereas F-statistics and adjusted R-squared were used for the OLS model. For the validation of the measurement of subjective well-being, life satisfaction and happiness measures were used. Importantly, the results from variated regression models are consistent, indicating a positive relationship between household energy consumption expenditure and the improvement of individuals’ subjective well-being. Regarding the model’s goodness of fit, the ***LR*** Chi-Square test with ordered logit and probit models, and the F-statistic in the OLS model are all statistically significant at 0.1%, which validates the regression model. As the consistency of the robustness results is derived from different models, the ordered logit model is applied in Table 2 (Panel B).

**Table 2:** Association between energy consumption expenditure and subjective well-being in high- and non-high-income countries.

Panel A	(1)	(2)	(3)	(4)
Variables	Life satisfaction	Happiness	Life satisfaction	Life satisfaction
Energy consumption expenditure	0.018***	0.008***	0.009***	0.009***
	(0.002)	(0.002)	(0.002)	(0.001)
Household equivalent income	0.309***	0.213***	0.310***	0.165***
	(0.008)	(0.009)	(0.008)	(0.005)
Other control variables	Yes	Yes	Yes	Yes
Country dummies	Yes	Yes	Yes	Yes
LR chi2(60)	11429.46	9698.53		10994.96
F statistic			197.54	
p-value	<0.0000	<0.0000	<0.0000	<0.0000
Obs.	84,913	84,913	84,913	84,913
Pseudo R-squared/Adjusted R-squared	0.0339	0.0463	0.123	0.0326
Model	Ordered logit	Ordered logit	Ols	Ordered probit

	High-income		Non-high-income	
Panel B	(1)	(2)	(3)	(4)
Variables	Life satisfaction	Happiness	Life satisfaction	Happiness
Energy consumption expenditure	0.010***	0.003	0.035***	0.015***
	(0.002)	(0.002)	(0.004)	(0.004)
Household equivalent income	0.405***	0.306***	0.250***	0.168***
	(0.014)	(0.015)	(0.011)	(0.011)
Other control variables	Yes	Yes	Yes	Yes
Country dummies	Yes	Yes	Yes	Yes
Obs.	37,572	37,572	47,341	47,341
Pseudo R-squared	0.0324	0.0446	0.0358	0.0495
	Ordered logit	Ordered logit	Ordered logit	Ordered logit

Standard errors are shown in parentheses. ***p < 0.01, **p < 0.05, *p < 0.1. Other control variables include education attainment, age, occupational status, household status, number of children, and gender dummy.

With the control variables being constant, energy consumption expenditure improves subjective well-being, including life satisfaction and happiness. The coefficients for the relationship of energy consumption with life satisfaction and with happiness are 0.018 and 0.008, respectively, and they are statistically significant at the 1% level; in other words, there is increased energy consumption for people who are satisfied with their lives and are happier. This is because electricity, water, gas, or gasoline are indispensable consumption goods in daily life. The results suggest that when policies lead to a reduction in the consumption of these goods at the household level, the life satisfaction of citizens is likely to decrease. When reducing energy consumption at the household level to reduce the emission of greenhouse gases, the conflicts of interest of individuals in these households (given that they derive life satisfaction from energy consumption) pose a challenge to policymakers; therefore, policymakers should devise strategies to improve both citizens’ living standards and environmental preservation.

Referring to the criteria developed by the World Bank, the standard classification of high-income nations and non-high-income nations is as follows. Based on the 2017 gross national income (GNI) per capita, the *World Bank List of Economies (June 2018)* presented the following criteria for nations to be classified as high-income and non-high-income nations, respectively: a GNI per capita of $12,056 or higher, and less than $12,056. According to this standard of classification, in this study, high-income nations comprise Japan, Singapore, Chile, Australia, the United States, Germany, the United Kingdom, France, Spain, Italy, Sweden, Canada, Netherlands, Greece, Hungary, Poland, and the Czech Republic, whereas non-high-income nations comprise Thailand, Malaysia, Indonesia, Vietnam, Philippines, Mexico, Venezuela, Brazil, Colombia, South Africa, India, Myanmar, Kazakhstan, Mongolia, Egypt, Russia, China, Turkey, Romania, and Sri Lanka.

Regarding the comparison of high- and non-high-income countries, energy consumption at the household level is more likely to lead to life satisfaction in non-high-income than in high-income countries. In high-income countries, the coefficients for the relationship of energy consumption with life satisfaction and with happiness are 0.010 and 0.003, respectively; these coefficients are 0.035 and 0.015, respectively, among non-high-income countries. Hence, in both high-income and non-high-income countries, an increase in energy consumption leads to an increase in life satisfaction; nonetheless, energy consumption is more crucial for households in non-high-income countries. Compared to the effect of energy consumption on satisfaction in high-income countries and non-high-income countries, individuals living in less urbanized countries appear more satisfied with energy consumption.

Table 3 presents the association between life satisfaction and energy consumption expenditure at the household level in each country by estimating Eq. (2) based on the ordered logit model for each country. There is a positive relationship between energy consumption expenditure and life satisfaction in 27 out of the 37 nations. For example, the coefficient of this relationship is 0.062 in Brazil, and is statistically significant at the 1% level. An increase in energy consumption expenditure positively impacts the life satisfaction of households in Brazil, meaning that individuals with greater energy expenditure tend to be satisfied with their lives. Similar results are found in other countries: Canada, Chile, China, Egypt, France, Germany, Greece, India, Indonesia, Italy, and Japan. As life satisfaction is a proxy of well-being, energy consumption is expected to increase when households can afford more energy to obtain higher life satisfaction. These results indicate that most of the developed and developing countries analyzed face a conflict of interest in addressing individuals’ life satisfaction and environment conservation goals; these countries include China and India that are home to large populations that have a positive desire for energy consumption.

**Table 3:** Relationship between energy expenditure and life satisfaction for each country.

Country name	Energy expenditure (Unit: $100)		Country name	Energy expenditure (Unit: $100)	
	Coeff.	(S.E.)		Coeff.	(S.E.)
Australia	0.007	(0.009)	Mongolia	0.214*	(0.129)
Brazil	0.062***	(0.018)	Myanmar	0.131***	(0.037)
Canada	0.022*	(0.012)	Netherlands	0.008	(0.011)
Chile	0.056***	(0.020)	Philippines	0.034**	(0.016)
China	0.155***	(0.009)	Poland	0.014	(0.012)
Colombia	0.030	(0.026)	Romania	0.024*	(0.013)
Czech	0.031	(0.031)	Russia	0.034	(0.034)
Egypt	0.741***	(0.123)	Singapore	−0.011	(0.009)
France	0.032***	(0.007)	South Africa	0.027*	(0.014)
Germany	0.036***	(0.007)	Spain	0.045***	(0.009)
Greece	0.039**	(0.017)	Sri Lanka	0.591***	(0.174)
Hungary	−0.014	(0.013)	Sweden	0.009	(0.012)
India	0.071***	(0.007)	Thailand	0.063***	(0.013)
Indonesia	0.069***	(0.012)	Turkey	0.046**	(0.021)
Italy	0.020**	(0.008)	United Kingdom	0.055***	(0.006)
Japan	0.021***	(0.005)	United States	0.035***	(0.003)
Kazakhstan	0.619***	(0.129)	Venezuela	0.041**	(0.019)
Malaysia	0.016	(0.021)	Vietnam	0.139***	(0.041)
Mexico	0.040*	(0.024)			

Standard errors are shown in parentheses. ***p < 0.01, **p < 0.05, *p < 0.1.

However, the association between life satisfaction and energy consumption expenditure at the household level was non-significant across some countries. In Australia, the coefficient of this association is positive but not statistically significant; hence, an increase in energy expenditure is not completely associated with life satisfaction at the household level here. Similar results are found in the Netherlands, Hungary, Sweden, Singapore, Poland, the Czech Republic, and Colombia. In these countries, energy consumption is at an adequate level, and additional energy consumption does not lead to higher life satisfaction. It may be that households consume an adequate amount of energy with their income and energy price.

Tables 4, 5, 6, and 7 display the determinant factors of household energy consumption in 37 nations by estimating the energy demand equation for each country using Eq. (3). The key energy consumption metric is the quantity of energy consumed (e.g., kWh) across the targeted households. Since price information is limited, transforming consumption expenditure into a quantity (e.g., kWh) is problematic. As explained earlier, this study adopted the energy demand equation.

**Table 4:** Household socioeconomic and demographic determinants of household energy consumption expenditure I.

Variables	Australia	Brazil	Canada	Chile	China	Colombia	Czech	Egypt	France	Germany
	(1)	(2)	(3)	(4)	(5)	(6)	(7)	(8)	(9)	(10)
	Energy	Energy	Energy	Energy	Energy	Energy	Energy	Energy	Energy	Energy
Household equivalent income	0.972***	2.901***	0.947***	2.785***	1.098***	2.931***	1.616***	2.876***	2.482***	1.442***
	(0.064)	(0.082)	(0.064)	(0.108)	(0.012)	(0.115)	(0.060)	(0.213)	(0.057)	(0.038)
Energy curtailment behavior	− 0.345	− 0.072	− 0.642*	0.082	0.027	− 0.348***	0.104	− 0.062	− 0.375	− 0.407*
	(0.369)	(0.106)	(0.332)	(0.162)	(0.019)	(0.126)	(0.115)	(0.114)	(0.273)	(0.212)
Buy energy saving product	− 0.763**	0.018	− 0.583*	− 0.069	− 0.044**	− 0.032	− 0.245***	− 0.041	0.144	− 0.168
	(0.316)	(0.097)	(0.309)	(0.180)	(0.019)	(0.129)	(0.093)	(0.093)	(0.296)	(0.194)
*Education attainment* (ref. junior school or lower)										
High school	− 0.003	− 0.021	− 1.035**	− 0.045	− 0.068	− 0.058	− 0.217*	− 0.096	− 0.529	− 1.052***
	(0.491)	(0.139)	(0.523)	(0.253)	(0.053)	(0.214)	(0.116)	(0.123)	(0.461)	(0.369)
Professional school	− 0.427	− 0.074	− 0.879	− 0.299	0.089	0.062	− 0.303*	− 0.160	− 0.413	− 1.154***
	(0.602)	(0.161)	(0.623)	(0.285)	(0.059)	(0.297)	(0.155)	(0.149)	(0.452)	(0.263)
College or university	− 0.428	− 0.244*	− 1.128**	− 0.307	− 0.027	0.066	− 0.387**	0.017	− 1.399***	− 0.813***
	(0.454)	(0.141)	(0.484)	(0.228)	(0.046)	(0.187)	(0.165)	(0.091)	(0.430)	(0.302)
Graduate school	− 0.781	− 0.208	− 1.117*	− 0.985**	0.057	− 0.638**	− 0.681***	− 0.114	− 1.618***	− 1.337***
	(0.597)	(0.227)	(0.627)	(0.382)	(0.060)	(0.282)	(0.139)	(0.106)	(0.495)	(0.388)
Age	− 0.088***	− 0.025***	− 0.069***	− 0.034***	− 0.003***	− 0.017***	− 0.007*	− 0.010**	− 0.148***	− 0.087***
	(0.013)	(0.004)	(0.012)	(0.008)	(0.001)	(0.005)	(0.004)	(0.004)	(0.011)	(0.008)
*Prices*										
Electricity price	0.475***	0.109	0.701***	0.221**	0.157***	0.204***	0.245***	0.068	0.628***	0.320***
	(0.166)	(0.094)	(0.152)	(0.105)	(0.012)	(0.078)	(0.060)	(0.054)	(0.173)	(0.115)
Gas price	0.104	0.019	0.077	0.006	0.097***	0.054	0.066***	− 0.091***	0.165**	0.184***
	(0.073)	(0.050)	(0.081)	(0.086)	(0.011)	(0.061)	(0.020)	(0.032)	(0.068)	(0.041)
Water price	0.188	0.078	0.009	0.144	− 0.008	0.202***	0.010	0.039	− 0.060	0.054
	(0.134)	(0.048)	(0.109)	(0.092)	(0.010)	(0.067)	(0.045)	(0.037)	(0.142)	(0.102)
Gasoline price	0.037	0.027	− 0.010	− 0.016	0.035***	− 0.011	0.068***	0.007	− 0.208**	− 0.075
	(0.064)	(0.033)	(0.094)	(0.046)	(0.008)	(0.029)	(0.023)	(0.015)	(0.103)	(0.058)
Other control variables	Yes	Yes	Yes	Yes	Yes	Yes	Yes	Yes	Yes	Yes
Observations	1387	1933	978	1026	19,023	987	1098	426	1661	2411
R-squared	0.262	0.455	0.332	0.467	0.360	0.460	0.463	0.543	0.602	0.477

Standard errors in parentheses. ***p < 0.01, **p < 0.05, *p < 0.1. Other control variables include occupational status, household status, number of children, and gender dummy.

**Table 5:** Household socioeconomic and demographic determinants of household energy consumption expenditure II.

Variables	Greece	Hungary	India	Indonesia	Italy	Japan	Kazakhstan	Malaysia	Mexico
	(11)	(12)	(13)	(14)	(15)	(16)	(17)	(18)	(19)
	Energy	Energy	Energy	Energy	Energy	Energy	Energy	Energy	Energy
Household equivalent income	1 435***	2.282***	3.134***	3.488***	1.389***	0.756***	2.295***	2.284***	2.680***
	(0.063)	(0.041)	(0.047)	(0.063)	(0.052)	(0.016)	(0.120)	(0.126)	(0.086)
Energy curtailment behavior	− 0.200	0.102	− 0.649***	0.332**	− 0.461**	− 0.547***	0.013	− 0.182	− 0.037
	(0.177)	(0.134)	(0.104)	(0.142)	(0.227)	(0.082)	(0.054)	(0.198)	(0.096)
Buy energy saving product	0.012	− 0.034	− 0.122	0.108	− 0.526**	0.037	− 0.058*	− 0.274	0.103
	(0.165)	(0.134)	(0.091)	(0.121)	(0.222)	(0.083)	(0.034)	(0.176)	(0.103)
*Education attainment* (ref. junior school or lower)									
High school	− 0.355	− 0.179	− 0.550**	− 0.240	− 0.466	− 0.362	− 0.095	− 0.972**	0.019
	(0.365)	(0.204)	(0.255)	(0.348)	(0.312)	(0.293)	(0.101)	(0.416)	(0.236)
professional school	− 0.223	− 0.127	− 0.867***	0.053	0.165	− 0.254	− 0.045	− 0.252	0.149
	(0.383)	(0.220)	(0.263)	(0.383)	(0.438)	(0.312)	(0.095)	(0.540)	(0.202)
College or university	− 0.144	− 0.242	− 0.726***	− 0.322	0.047	− 0.586**	− 0.071	− 0.321	0.067
	(0.341)	(0.162)	(0.177)	(0.331)	(0.418)	(0.286)	(0.096)	(0.243)	(0.190)
Graduate school	0.306	− 0.720**	− 0.700***	− 0.779**	− 0.309	− 1.021***	− 0.120	− 0.649*	0.076
	(0.388)	(0.304)	(0.180)	(0.385)	(0.366)	(0.315)	(0.151)	(0.362)	(0.242)
Age	− 0.008	− 0.021***	− 0.032***	− 0.006	− 0.057***	− 0.037***	− 0.004**	− 0.045***	− 0.020***
	(0.009)	(0.006)	(0.005)	(0.007)	(0.009)	(0.004)	(0.001)	(0.011)	(0.004)
*Prices*									
Electricity price	0.027	0.362***	0.186***	0.200**	0.720***	0.202***	0.135***	0.185*	0.336***
	(0.126)	(0.096)	(0.059)	(0.080)	(0.163)	(0.052)	(0.030)	(0.111)	(0.058)
Gas price	− 0.008	0.054	0.087	− 0.045	0.158	0.060***	− 0.007	0.056	− 0.019
	(0.041)	(0.044)	(0.055)	(0.073)	(0.124)	(0.023)	(0.030)	(0.082)	(0.064)
Water price	− 0.076	− 0.028	0.134***	− 0.030	0.207*	0.124***	− 0.062**	0.247**	0.008
	(0.076)	(0.078)	(0.042)	(0.057)	(0.117)	(0.043)	(0.028)	(0.099)	(0.047)
Gasoline price	0.024	0.023	0.070***	0.095	− 0.092	0.169***	0.011	0.076	0.011
	(0.082)	(0.037)	(0.026)	(0.063)	(0.103)	(0.023)	(0.008)	(0.054)	(0.037)
Other control variables	Yes	Yes	Yes	Yes	Yes	Yes	Yes	Yes	Yes
Observations	1050	1109	4682	2023	1728	8498	755	997	1474
R-squared	0.405	0.750	0.539	0.622	0.380	0.268	0.461	0.313	0.468

Standard errors in parentheses. ***p < 0.01, **p < 0.05, *p < 0.1. Other control variables include occupational status, household status, number of children, and gender dummy.

**Table 6:** Household socioeconomic and demographic determinants of household energy consumption expenditure III.

Variables	Mongolia	Myanmar	Netherlands	Philippines	Poland	Romania	Russia	Singapore	SouthAfrica	Spain
	(20)	(21)	(22)	(23)	(24)	(25)	(26)	(27)	(28)	(29)
	Energy	Energy	Energy	Energy	Energy	Energy	Energy	Energy	Energy	Energy
Household equivalent income	3.448***	1.525***	1.461***	3.613***	2.079***	1.782***	1.686***	1.603***	2.262***	2.013***
	(0.241)	(0.043)	(0.070)	(0.097)	(0.046)	(0.043)	(0.056)	(0.136)	(0.100)	(0.053)
Energy curtailment behavior	0.018	0.002	− 0.744**	− 0.103	− 0.265**	− 0.126	− 0.073*	− 0.056	0.109	− 0.324
	(0.056)	(0.065)	(0.314)	(0.179)	(0.130)	(0.158)	(0.044)	(0.943)	(0.316)	(0.203)
Buy energy saving product	0.020	0.005	0.223	− 0.093	0.290**	− 0.054	0.048	− 0.872	0.143	0.305
	(0.095)	(0.065)	(0.317)	(0.130)	(0.116)	(0.164)	(0.048)	(0.854)	(0.235)	(0.196)
*Education attainment* (ref. junior school or lower)										
High school	0.044	0.063	− 1.049**	− 0.247	− 0.157	0.054	− 0.122	− 2.232	− 0.952**	− 0.816**
	(0.081)	(0.076)	(0.502)	(0.334)	(0.200)	(0.351)	(0.140)	(1.703)	(0.474)	(0.320)
Professional school	0.255**	− 0.189	− 1.095**	− 0.091	− 0.076	− 0.115	− 0.141	− 0.906	−	− 0.428
	(0.106)	(0.363)	(0.451)	(0.433)	(0.220)	(0.492)	(0.123)	(1.686)	−	(0.308)
College or university	0.046	− 0.055	− 1.263***	− 0.291	− 0.228	0.157	− 0.198*	− 2.021*	− 1.435***	− 1.090***
	(0.072)	(0.077)	(0.415)	(0.260)	(0.211)	(0.338)	(0.120)	(1.190)	(0.466)	(0.294)
Graduate school	0.071	0.060	− 1.261**	− 0.801**	− 0.625***	− 0.065	− 0.187	− 2.855*	0.143	− 0.950**
	(0.131)	(0.261)	(0.559)	(0.356)	(0.194)	(0.370)	(0.143)	(1.630)	(0.672)	(0.384)
Age	− 0.007***	− 0.003	− 0.123***	− 0.014**	− 0.024***	− 0.011	− 0.015***	− 0.084**	− 0.048***	− 0.068***
	(0.002)	(0.003)	(0.012)	(0.006)	(0.005)	(0.007)	(0.002)	(0.040)	(0.010)	(0.008)
*Prices*										
Electricity price	0.063**	− 0.012	0.124	0.137	0.258***	0.427***	0.135***	1.285*	0.160	− 0.329**
	(0.027)	(0.038)	(0.175)	(0.093)	(0.075)	(0.122)	(0.031)	(0.678)	(0.197)	(0.143)
Gas price	0.008	0.028*	− 0.065	0.042	0.142***	0.098	0.023*	0.249	0.055	0.087
	(0.012)	(0.016)	(0.128)	(0.072)	(0.038)	(0.066)	(0.012)	(0.412)	(0.058)	(0.053)
Water price	0.047*	− 0.040	0.323**	0.034	− 0.045	− 0.141	0.040	− 0.212	0.166	0.221**
	(0.025)	(0.028)	(0.161)	(0.071)	(0.062)	(0.086)	(0.025)	(0.644)	(0.110)	(0.104)
Gasoline price	0.035***	0.065***	0.011	0.006	0.015	0.020	0.026**	− 0.039	− 0.038	− 0.140*
	(0.013)	(0.015)	(0.085)	(0.054)	(0.032)	(0.049)	(0.012)	(0.196)	(0.049)	(0.073)
Other control variables	Yes	Yes	Yes	Yes	Yes	Yes	Yes	Yes	Yes	Yes
Observations	443	1057	936	1451	1725	1173	2026	482	932	1798
R-squared	0.460	0.601	0.492	0.510	0.569	0.612	0.365	0.264	0.417	0.502

Standard errors in parentheses. ***p < 0.01, **p < 0.05, *p < 0.1. Other control variables include occupational status, household status, number of children, and gender dummy.

**Table 7:** Household socioeconomic and demographic determinants of household energy consumption expenditure IV.

Variables	Sri Lanka	Sweden	Thailand	Turkey	United Kingdom	United States	Venezuela	Vietnam
	(30)	(31)	(32)	(33)	(34)	(35)	(36)	(37)
	Energy	Energy	Energy	Energy	Energy	Energy	Energy	Energy
Household equivalent income	3.111***	1.607***	3.406***	2.278***	1.554***	1.332***	2.319***	2.311***
	(0.267)	(0.078)	(0.115)	(0.045)	(0.053)	(0.027)	(0.149)	(0.095)
Energy curtailment behavior	− 0.070	− 0.349	− 0.295	− 0.299***	− 0.583**	− 0.956***	0.316	− 0.058
	(0.043)	(0.323)	(0.264)	(0.076)	(0.258)	(0.165)	(0.260)	(0.069)
Buy energy saving product	− 0.078	− 0.005	− 0.022	0.162**	− 0.343	− 0.179	0.171	0.015
	(0.048)	(0.392)	(0.223)	(0.067)	(0.239)	(0.157)	(0.249)	(0.055)
*Education attainment (ref. junior school or lower)*								
High school	0.027	− 1.246***	− 0.141	− 0.493***	− 0.392	− 1.881***	0.288	0.179
	(0.048)	(0.461)	(0.520)	(0.138)	(0.440)	(0.275)	(0.492)	(0.127)
Professional school	0.039	− 0.982*	− 0.184	− 0.456**	− 0.528	− 1.553***	− 0.696	0.277*
	(0.259)	(0.596)	(0.585)	(0.180)	(0.602)	(0.362)	(0.625)	(0.165)
College or university	0.043	− 1.435***	− 0.323	− 0.634***	− 0.931**	− 2.292***	− 0.176	0.206*
	(0.126)	(0.469)	(0.412)	(0.130)	(0.412)	(0.267)	(0.445)	(0.108)
Graduate school	0.057	− 1.094*	− 0.688	− 0.700***	− 0.800	− 1.342***	0.178	− 0.120
	(0.136)	(0.659)	(0.489)	(0.164)	(0.488)	(0.310)	(0.563)	(0.146)
Age	− 0.005**	− 0.048***	− 0.018	− 0.018***	− 0.116***	− 0.119***	− 0.013	− 0.005
	(0.002)	(0.013)	(0.011)	(0.004)	(0.010)	(0.006)	(0.012)	(0.004)
*Prices*								
Electricity price	0.039	0.321**	0.429***	0.112**	0.468***	0.829***	0.386***	0.028
	(0.026)	(0.153)	(0.150)	(0.054)	(0.153)	(0.085)	(0.122)	(0.038)
Gas price	0.050***	0.380***	0.196*	− 0.011	0.134*	0.197***	0.256**	0.038
	(0.016)	(0.076)	(0.108)	(0.031)	(0.075)	(0.044)	(0.115)	(0.033)
Water price	0.012	0.353***	0.006	0.079**	0.063	0.206***	0.150	0.015
	(0.015)	(0.120)	(0.127)	(0.035)	(0.127)	(0.062)	(0.116)	(0.031)
Gasoline price	0.009	− 0.047	0.087	− 0.020	0.178***	0.045	0.112	− 0.014
	(0.014)	(0.088)	(0.121)	(0.032)	(0.048)	(0.055)	(0.091)	(0.036)
Other control variables	Yes	Yes	Yes	Yes	Yes	Yes	Yes	Yes
Observations	461	864	1064	1931	2144	8590	696	1467
R-squared	0.334	0.440	0.504	0.612	0.409	0.374	0.339	0.348

Standard errors in parentheses. ***p < 0.01, **p < 0.05, *p < 0.1. Other control variables include occupational status, household status, number of children, and gender dummy.

There are positive relationships between energy consumption expenditure at the household level and household income across countries. If the coefficients for household income are positive and statistically significant, this means that energy consumption expenditure at the household level would increase with an increase in household income ensuing from economic development in the country, ceteris paribus. The positive coefficients for the association between energy consumption expenditure and household income range from 0.756 (Japan) to 3.613 (the Philippines) in our sample, indicating that an additional 10,000 USD would lead to an additional energy consumption expenditure at the household level of approximately 17.3% (Japan) – 445% (Mongolia). The number is calculated using the magnitude of the coefficient/energy consumption expenditure. The results also show that homeowners tend to consume more energy than renters in Australia, Brazil, Canada, Chile, China, Colombia, Germany, India, Italy, Japan, Malaysia, Mexico, Russia, the United States, and Vietnam. This indicates that if individuals live in their own houses, the household energy consumption expenditure tends to be higher owing to the wealth effect, as energy is a normal consumption good. Overall, the wealth effect on energy consumption expenditure at the household level is increasing in our sample, and with economic development, energy consumption may increase.

The following factors are confirmed to reduce energy consumption at the household level: (1) energy-curtailment behavior regarding electricity, (2) higher education, and (3) age. The energy-saving effect is confirmed in households. In Canada, the coefficient of energy-saving behaviors is -0.642, indicating that households consume 12.5% less energy when they adopt both energy curtailment behavior and non-saving groups (64.2/513). The Canadian household average energy consumption is 513 USD. Similar results are seen in Colombia, Germany, India, Indonesia, Italy, Japan, the Netherlands, Poland, Russia, Turkey, the United Kingdom, and the United States. The magnitude of the effect of energy curtailment behavior ranged from 6.4% (Russia) to 32% (India) less energy consumption expenditure. Hence, energy-saving behaviors have a favorable effect on environmentally preferable outcomes. By contrast, households in Indonesia save electricity as they tend to spend more on purchasing energy.

Individuals with higher education tend to save energy in 23 out of the 37 nations. For instance, the coefficient for individuals with university-level education is -2.292 and statistically significant at the 1% level. This suggests that households with individuals who have university-level education have less energy consumption expenditure than households with individuals with junior high school or lower levels of education. Similar results are seen in Brazil, Canada, Chile, Colombia, the Czech Republic, France, Germany, Hungary, India, Indonesia, Japan, Malaysia, the Netherlands, the Philippines, Poland, Russia, Singapore, South Africa, Spain, Sweden, Turkey, the United Kingdom, and the United States. Encouraging households to engage in energy curtailment behaviors and higher educational attainment may lead to environment-friendly outcomes.

Surprisingly, purchasing energy-saving household products has a limited effect on reducing energy consumption expenditure at the household level. The coefficients for purchasing energy-saving household products are negative, ranging between -0.044 and -0.763, and are statistically significant in Australia, Canada, the Czech Republic, Italy, and Kazakhstan. Hence, the purchase of these products in these five countries decreases energy expenditure from 2.9% (China) to 14% (Australia). However, the relationship between energy consumption expenditure at the household level and purchasing energy-saving household products is non-significant in the other countries. Moreover, in Poland and Turkey, households that purchase these products consume more energy than those that do not. Therefore, purchasing energy-saving household products has a limited contribution to energy saving at the household level.

The findings also show that older individuals tend to have lower energy consumption. The coefficients for the age variable are negative and statistically significant in 30 countries (out of 37). The effect of age on energy consumption expenditure ranges between -0.003 and -0.148, indicating that as the average age of individuals increases by one year, their monthly energy consumption expenditure reduces from 0.3–14.8 USD. This may be because older individuals are more likely to live frugally.

## Discussion

In this study, we identify whether greenhouse gas emissions at the household level can be reduced by reducing energy consumption expenditure at the household level. To confirm this, (1) we investigate the relationship between energy consumption expenditure at the household level and life satisfaction, and (2) examine the effect of energy-saving behavior on reducing energy consumption expenditure at the household level, and find the following trends.

First, the evidence shows a positive association between energy consumption expenditure at the household level and subjective well-being; specifically, an increase in the former is expected to increase the likelihood of people being satisfied with their lives in 27 (out of 37) of the surveyed countries, including China and the United States. These results corroborate the evidence in prior research^32,33,35,41–43^, and that endeavoring to support the achievement of environmental conservation goals through reducing energy-related greenhouse gas emissions at the household level is likely to pose many challenges. Compared to the effect of energy consumption on satisfaction in high-income countries and non-high-income countries, individuals living in less urbanized countries appear more satisfied with energy consumption.

Second, (1) energy curtailment behaviors, (2) higher education, and (3) age show environmentally favorable effects on energy consumption expenditure at the household level, consistent with prior studies^29–40^. A policy implication of this finding is that encouraging energy-saving behaviors, reducing population, promoting higher educational attainment, and adopting frugal lifestyles (often related to older adults) may facilitate sustainable energy consumption and general well-being. However, the purchase of energy-saving products has a limited effect on energy consumption expenditure, and policymakers and corporations may need to make greater efforts in the research and development of energy-saving electronic products.

Globally, both energy prices and household income have been increasing in recent decades. Increasing energy prices are believed to reduce household energy consumption. However, increased household wealth allows households to consume more energy. In recent decades, owing to economic growth, a standard household can afford greater amounts of energy. Moreover, when household income increases, the household income spent on energy increases.

Based on previous studies on household energy consumption, energy is confirmed as a normal good^44,62–64^. Since energy is a normal good, an increase in energy consumption is more likely to lead to greater well-being. When household income increases, the share of normal goods (energy in this case) also increases in the overall household budget. If energy were an inferior good, then as household income increases, the expenditure on energy would decrease under the new optimized consumption choices. The results are consistent with previous studies showing that wealthy households consume more energy^65,66^, whereas prior studies investigated the link between economic growth and CO2 at the country level. Therefore, achieving a reduction in energy consumption, and simultaneously, carbon emissions, might be difficult under a household’s rational decision-making.

At the household level, energy-saving behavior and purchasing energy-saving products will reduce energy consumption expenditure. We found that either energy curtailment behavior or purchasing energy-saving electronic products will have a limited effect when household energy is mainly sourced from fossil fuels; relying on energy-saving goods will have a limited effect when pursuing the goal of carbon emission reduction. As the carbon neutral goal should be achieved until the 2050s, household energy sourced from renewable energy or other non-fossil fuels might be a good alternative to reduce the household sector’s carbon emissions. To help address the climate change issue caused by emissions at the household consumer level, various governments are enacting legislation to reduce such emissions.

However, according to this study’s results, when fossil fuels are the primary energy source, reducing greenhouse gas emissions by decreasing energy consumption at the household level might prove difficult. Fortunately, the renewable energy sector is developing rapidly. This study encourages stakeholders to seek alternatives, such as nuclear power or preferably renewable energy, to reduce greenhouse gas emissions at the household level.

The study has the following limitations. First, the sample selection approach could result in selection bias in the survey. We primarily relied on an internet survey approach, which might have skewed the sample toward wealthy and well-educated households. To address this issue, face-to-face surveys were conducted in Mongolia, Myanmar, Egypt, Kazakhstan, Sri Lanka, Indonesia, India, and Malaysia to confirm the robustness of the main results. However, sample selection bias might still exist. Future studies should use comprehensive datasets to investigate household energy consumption, well-being, and environmental sustainability. Second, as the information on energy prices is limited, households’ expenditure on energy is used to measure household energy consumption in each country. We assume that the greater the amount spent on energy, the greater the amount of energy consumed by households. This assumption could potentially cause bias in the results. Therefore, future studies should use comprehensive price information with accurate household energy consumption information.

## Conclusion

Energy consumption is considered a major contributor to climate change due to CO2 emissions. Therefore, energy consumption at the household level has caught the attention of numerous researchers and policymakers. Our study raises two questions: how does energy consumption influence life satisfaction? How can energy consumption at the household level be reduced? By conducting a large-scale survey using both internet-based and face-to-face approaches across 37 nations on 6 continents (comprising approximately 73% of the world population), this study demonstrates the relationship between life satisfaction and energy consumption expenditure at the household level, as well as the determinants of energy consumption behavior in households.

## Supplementary Information

Supplementary Information.

## Data Availability

The datasets used and analyzed during the current study are available from the corresponding author on reasonable request. The questionnaire and data are available upon reasonable request to the authors.
