# Supplementary material for: Household energy-saving behavior, its consumption, and life satisfaction in 37 countries
Source: Sci Rep. 2023 Jan 25;13:1382. doi: 10.1038/s41598-023-28368-8 (PMC9876990; doi:10.1038/s41598-023-28368-8)
Supplement: Supplementary file 1 — Supplementary Information. [file 41598_2023_28368_MOESM1_ESM.docx]

**Appendix**

Figure A1: Distribution of the households’ energy expenditure

Data sources: Original survey from 37 nations

**Table A1. Descriptive statistics**

| Variable | Obs | Percent/Mean | Std. Dev. | Min | Max |
| --- | --- | --- | --- | --- | --- |
| Life satisfaction | 100,956 | 6.65 | 1.91 | 0 | 10 |
| Happiness | 100,956 | 3.89 | 0.95 | 1 | 5 |
| Energy expenditure (100USD) | 84,913 | 3.10 | 4.88 | 0.00 | 97.33 |
| Household equivalent income | 92,128 | 1.93 | 2.25 | 0.00 | 27.13 |
| Save electricity | 100,956 | 0.61 | 0.49 | 0 | 1 |
| Buy energy saving product | 100,956 | 0.47 | 0.50 | 0 | 1 |
| *Education attainment dummy variables* |  |  |  |  |  |
| junior school or lower | 100,956 | 8% |  |  |  |
| high school | 100,956 | 19% |  |  |  |
| professional school | 100,956 | 10% |  |  |  |
| college or university | 100,956 | 50% |  |  |  |
| graduate school | 100,956 | 11% |  |  |  |
| Age | 100,956 | 42.73 | 15.01 | 18 | 99 |
| *Subjective price* |  |  |  |  |  |
| electricity price | 100,238 | 4.94 | 1.01 | 0 | 6 |
| gas price | 100,238 | 4.29 | 1.69 | 0 | 6 |
| water price | 100,238 | 4.43 | 1.19 | 0 | 6 |
| gasoline price | 100,238 | 4.50 | 1.78 | 0 | 6 |
| *Occupational status dummy variables* |  |  |  |  |  |
| unemployed | 100,956 | 9% |  |  |  |
| company owner | 100,956 | 2% |  |  |  |
| government employee | 100,956 | 3% |  |  |  |
| professional employee | 100,956 | 3% |  |  |  |
| full time employee | 100,956 | 43% |  |  |  |
| part time employee | 100,956 | 8% |  |  |  |
| self employed | 100,956 | 8% |  |  |  |
| student | 100,956 | 6% |  |  |  |
| housewife/househusband | 100,956 | 9% |  |  |  |
| other | 100,956 | 9% |  |  |  |
| *House status dummy variables* |  |  |  |  |  |
| house owner | 100,956 | 71% |  |  |  |
| house renter | 100,956 | 4% |  |  |  |
| *Number of children dummy variables* |  |  |  |  |  |
| one child | 100,956 | 28% |  |  |  |
| two children | 100,956 | 24% |  |  |  |
| three children or more | 100,956 | 12% |  |  |  |
| female dummy | 100,956 | 50% |  |  |  |

Data sources: The internet survey conducted in the current study.

**Table A2. Country list**

| Country name | Start time | End time | Survey type | Observation |
| --- | --- | --- | --- | --- |
| Japan | 2015/7/14 | 2015/8/5 | Internet | 11167 |
| Thailand | 2015/7/18 | 2015/7/23 | Internet | 1127 |
| Malaysia | 2015/7/23 | 2015/7/29 | Internet | 1106 |
| Indonesia | 2015/7/18 | 2015/7/23 | Internet | 2210 |
| Singapore | 2015/7/15 | 2015/7/21 | Internet | 587 |
| Vietnam | 2015/7/18 | 2015/7/28 | Internet | 1541 |
| Philippines | 2015/7/15 | 2015/7/22 | Internet | 1686 |
| Mexico | 2015/7/24 | 2015/7/27 | Internet | 1678 |
| Venezuela | 2015/7/24 | 2015/8/5 | Internet | 827 |
| Chile | 2015/7/24 | 2015/7/28 | Internet | 1192 |
| Brazil | 2015/7/23 | 2015/7/26 | Internet | 2298 |
| Colombia | 2015/7/24 | 2015/7/27 | Internet | 1115 |
| South Africa | 2015/7/15 | 2015/7/23 | Internet | 1123 |
| India | 2015/7/25 | 2015/8/11 | Internet | 5200 |
| Myanmar | 2015/7/6 | 2015/8/10 | Face-to-face | 1083 |
| Indonesia | 2015/6/30 | 2015/8/11 | Face-to-face | 202 |
| Vietnam | 2015/6/24 | 2015/6/30 | Face-to-face | 200 |
| India | 2015/7/21 | 2015/8/25 | Face-to-face | 1500 |
| Kazakhstan | 2015/8/25 | 2015/9/24 | Face-to-face | 1000 |
| Mongolia | 2015/8/19 | 2015/9/3 | Face-to-face | 500 |
| Egypt | 2015/9/14 | 2015/10/27 | Face-to-face | 1016 |
| Russia | 2015/8/31 | 2015/9/14 | Internet | 2221 |
| China | 2016/1/12 | 2016/2/29 | Internet | 20744 |
| Australia | 2016/2/10 | 2016/2/22 | Internet | 2029 |
| United States | 2016/8/16 | 2016/8/28 | Internet | 10683 |
| Germany | 2016/8/26 | 2016/9/7 | Internet | 3165 |
| United Kingdom | 2016/8/16 | 2016/8/28 | Internet | 2993 |
| France | 2016/8/26 | 2016/9/7 | Internet | 2138 |
| Spain | 2016/8/26 | 2016/9/7 | Internet | 2116 |
| Italy | 2016/8/29 | 2016/9/10 | Internet | 2106 |
| Sweden | 2016/8/31 | 2016/9/12 | Internet | 1330 |
| Canada | 2016/9/1 | 2016/9/13 | Internet | 1333 |
| Netherlands | 2016/8/29 | 2016/9/10 | Internet | 1371 |
| Greece | 2016/8/31 | 2016/9/12 | Internet | 1382 |
| Turkey | 2017/3/7 | 2017/3/20 | Internet | 2120 |
| Hungary | 2017/3/8 | 2017/3/15 | Internet | 1354 |
| Poland | 2017/3/8 | 2017/3/17 | Internet | 2227 |
| Czech Republic | 2017/3/8 | 2017/3/16 | Internet | 1400 |
| Romania | 2017/3/8 | 2017/3/18 | Internet | 1386 |
| Sri Lanka | 2017/3/9 | 2017/3/30 | Face to face | 500 |

**Table A3**

**Table A3:** Relationship between energy expenditure and life satisfaction for each country (two-stage least square regression)

| Country name | Energy expenditure (Unit: $100) |  |
| --- | --- | --- |
|  | Coeff. | (S.E.) |
| Australia | 0.106*** | (0.024) |
| Brazil | 0.125*** | (0.028) |
| Canada | 0.120*** | (0.029) |
| Chile | 0.104*** | (0.028) |
| China | 0.375*** | (0.014) |
| Colombia | 0.114*** | (0.036) |
| Czech | 0.260*** | (0.051) |
| Egypt | 1.167*** | (0.208) |
| France | 0.039*** | (0.008) |
| Germany | 0.094*** | (0.011) |
| Greece | 0.059** | (0.029) |
| Hungary | 0.012 | (0.017) |
| India | 0.055*** | (0.009) |
| Indonesia | 0.071*** | (0.011) |
| Italy | 0.076*** | (0.015) |
| Japan | 0.273*** | (0.013) |
| Kazakhstan | 1.112*** | (0.217) |
| Malaysia | 0.089** | (0.038) |
| Mexico | 0.077*** | (0.028) |
| Mongolia | 0.430* | (0.231) |
| Myanmar | 0.211*** | (0.043) |
| Netherlands | 0.060*** | (0.017) |
| Philippines | 0.062*** | (0.020) |
| Poland | 0.064*** | (0.018) |
| Romania | 0.050*** | (0.015) |
| Russia | 0.441*** | (0.065) |
| Singapore | 0.015 | (0.020) |
| SouthAfrica | 0.111*** | (0.027) |
| Spain | 0.097*** | (0.012) |
| SriLanka | 1.113*** | (0.335) |
| Sweden | 0.083*** | (0.021) |
| Thailand | 0.083*** | (0.017) |
| Turkey | 0.078*** | (0.026) |
| United Kingdom | 0.111*** | (0.013) |
| United States | 0.085*** | (0.006) |
| Venezuela | 0.074* | (0.045) |
| Vietnam | 0.239*** | (0.068) |

**Table A4: Relationship of the household income and energy-friendly behavior and household energy consumption expenditure**

| Country name | Ln(Household equivalent income) | | Save electricity energy | | Buy energy saving product | |
| --- | --- | --- | --- | --- | --- | --- |
|  | Coeff. | (S.E.) | Coeff. | (S.E.) | Coeff. | (S.E.) |
| Australia | 3.276*** | (0.233) | -0.238 | (0.373) | -0.783** | (0.319) |
| Brazil | 1.665*** | (0.059) | -0.115 | (0.115) | 0.039 | (0.105) |
| Canada | 3.524*** | (0.225) | -0.601* | (0.328) | -0.703** | (0.306) |
| Chile | 2.019*** | (0.093) | 0.133 | (0.172) | -0.291 | (0.191) |
| China | 0.866*** | (0.012) | 0.037* | (0.021) | -0.051** | (0.021) |
| Colombia | 1.172*** | (0.063) | -0.331** | (0.140) | -0.096 | (0.143) |
| Czech | 1.244*** | (0.062) | 0.056 | (0.127) | -0.257** | (0.103) |
| Egypt | 0.555*** | (0.043) | -0.029 | (0.116) | -0.062 | (0.095) |
| France | 5.426*** | (0.223) | -0.724** | (0.346) | 0.254 | (0.375) |
| Germany | 4.562*** | (0.138) | -0.577*** | (0.222) | -0.393* | (0.204) |
| Greece | 2.677*** | (0.104) | -0.289* | (0.170) | -0.130 | (0.159) |
| Hungary | 2.533*** | (0.117) | 0.245 | (0.220) | -0.092 | (0.220) |
| India | 1.683*** | (0.041) | -0.789*** | (0.125) | -0.211* | (0.110) |
| Indonesia | 2.061*** | (0.079) | -0.093 | (0.194) | -0.157 | (0.167) |
| Italy | 3.756*** | (0.155) | -0.574** | (0.233) | -0.502** | (0.228) |
| Japan | 2.476*** | (0.056) | -0.607*** | (0.083) | 0.013 | (0.084) |
| Kazakhstan | 0.454*** | (0.029) | 0.034 | (0.057) | -0.059 | (0.037) |
| Malaysia | 1.895*** | (0.120) | -0.131 | (0.205) | -0.295 | (0.182) |
| Mexico | 1.453*** | (0.052) | -0.024 | (0.100) | 0.154 | (0.108) |
| Mongolia | 0.531*** | (0.048) | 0.032 | (0.060) | 0.044 | (0.102) |
| Myanmar | 1.257*** | (0.060) | 0.113 | (0.081) | 0.087 | (0.081) |
| Netherlands | 4.695*** | (0.282) | -0.782** | (0.335) | 0.255 | (0.337) |
| Philippines | 1.828*** | (0.074) | -0.291 | (0.210) | -0.123 | (0.153) |
| Poland | 2.110*** | (0.083) | -0.343** | (0.164) | 0.388*** | (0.146) |
| Romania | 2.211*** | (0.085) | -0.250 | (0.197) | -0.039 | (0.205) |
| Russia | 0.930*** | (0.036) | -0.071 | (0.046) | 0.023 | (0.050) |
| Singapore | 5.894*** | (0.577) | -0.014 | (0.971) | -1.172 | (0.881) |
| SouthAfrica | 2.178*** | (0.120) | 0.209 | (0.338) | -0.037 | (0.252) |
| Spain | 3.900*** | (0.154) | -0.549** | (0.235) | 0.329 | (0.227) |
| SriLanka | 0.548*** | (0.046) | -0.078* | (0.043) | -0.072 | (0.047) |
| Sweden | 3.609*** | (0.236) | -0.472 | (0.351) | -0.091 | (0.426) |
| Thailand | 3.067*** | (0.149) | -0.296 | (0.302) | -0.226 | (0.255) |
| Turkey | 1.047*** | (0.041) | -0.459*** | (0.100) | 0.228** | (0.088) |
| United Kingdom | 4.298*** | (0.187) | -0.735*** | (0.273) | -0.443* | (0.253) |
| United States | 4.471*** | (0.095) | -1.164*** | (0.167) | -0.225 | (0.158) |
| Venezuela | 2.112*** | (0.139) | 0.288 | (0.262) | 0.329 | (0.250) |
| Vietnam | 0.773*** | (0.037) | -0.136* | (0.072) | 0.032 | (0.057) |

Note: According to the demand equation theory [52-26], different type of energy demand equation is applied, whereas regressing the energy consumption expenditure on the Ln(household equivalent income) is applied in the robustness check. Standard errors are in parentheses. *** p<0.01, ** p<0.05, * p<0.1. Other control variables include education attainment, age, subjective price of the electricity, gas, water, and gasoline, occupational status, household status, number of children, and gender dummy.

|  | Energy consumption (100 USD) | Household equivalent income | | Save electricity energy | | Buy energy saving product | |
| --- | --- | --- | --- | --- | --- | --- | --- |
|  |  | Coeff. | % | Coeff. | % | Coeff. | % |
| Australia | 5.47 | 0.972 | 17.77 |  |  | -0.763 | -13.95 |
| Brazil | 1.84 | 2.901 | 157.59 |  |  |  |  |
| Canada | 5.13 | 0.947 | 18.46 | -0.642 | -12.52 | -0.583 | -11.37 |
| Chile | 3.01 | 2.785 | 92.60 |  |  |  |  |
| China | 1.52 | 1.098 | 72.28 |  |  | -0.044 | -2.90 |
| Colombia | 1.71 | 2.931 | 170.96 | -0.348 | -20.30 |  |  |
| Czech | 1.90 | 1.616 | 85.22 |  |  | -0.245 | -12.92 |
| Egypt | 0.66 | 2.876 | 432.75 |  |  |  |  |
| France | 5.33 | 2.482 | 46.55 |  |  |  |  |
| Germany | 5.51 | 1.442 | 26.18 | -0.407 | -7.39 |  |  |
| Greece | 3.37 | 1.435 | 42.56 |  |  |  |  |
| Hungary | 1.82 | 2.282 | 125.46 |  |  |  |  |
| India | 2.02 | 3.134 | 155.14 | -0.649 | -32.13 |  |  |
| Indonesia | 1.58 | 3.488 | 220.85 | 0.332 | 21.02 |  |  |
| Italy | 5.21 | 1.389 | 26.68 | -0.461 | -8.86 | -0.526 | -10.10 |
| Japan | 4.38 | 0.756 | 17.28 | -0.547 | -12.50 |  |  |
| Kazakhstan | 0.62 | 2.295 | 371.38 |  |  | -0.058 | -9.39 |
| Malaysia | 2.68 | 2.284 | 85.17 |  |  |  |  |
| Mexico | 1.98 | 2.68 | 135.36 |  |  |  |  |
| Mongolia | 0.77 | 3.448 | 444.92 |  |  |  |  |
| Myanmar | 0.47 | 1.525 | 322.61 |  |  |  |  |
| Netherlands | 4.83 | 1.461 | 30.23 | -0.744 | -15.39 |  |  |
| Philippines | 1.83 | 3.613 | 197.64 |  |  |  |  |
| Poland | 2.00 | 2.079 | 103.90 | -0.265 | -13.24 | 0.29 | 14.49 |
| Romania | 1.88 | 1.782 | 94.65 |  |  |  |  |
| Russia | 1.15 | 1.686 | 147.16 | -0.073 | -6.37 |  |  |
| Singapore | 7.48 | 1.603 | 21.43 |  |  |  |  |
| SouthAfrica | 2.90 | 2.262 | 78.12 |  |  |  |  |
| Spain | 4.27 | 2.013 | 47.15 |  |  |  |  |
| SriLanka | 0.50 | 3.111 | 619.64 |  |  |  |  |
| Sweden | 4.85 | 1.607 | 33.13 |  |  |  |  |
| Thailand | 3.85 | 3.406 | 88.39 |  |  |  |  |
| Turkey | 1.02 | 2.278 | 223.12 | -0.299 | -29.29 | 0.162 | 15.87 |
| UnitedKingdom | 5.16 | 1.554 | 30.09 | -0.583 | -11.29 |  |  |
| UnitedStates | 7.02 | 1.332 | 18.98 | -0.956 | -13.62 |  |  |
| Venezuela | 3.69 | 2.319 | 62.92 |  |  |  |  |
| Vietnam | 1.17 | 2.311 | 197.75 |  |  |  |  |

**Note: The coefficient is derived from Table 3 to table 6. The effect (%) is derived by magnitudes of the coefficient/household energy consumption. The statistically insignificant coefficients are omitted.**
